# The omega-3 polyunsaturated fatty acid docosahexaenoic acid inhibits proliferation and progression of non-small cell lung cancer cells through the reactive oxygen species-mediated inactivation of the PI3K /Akt pathway

**DOI:** 10.1186/s12944-017-0474-x

**Published:** 2017-05-03

**Authors:** Yuanqin Yin, Chengguang Sui, Fandong Meng, Ping Ma, Youhong Jiang

**Affiliations:** Cancer Institute, First Affiliated Hospital, China Medical University, Shenyang, Liaoning 110001 China

**Keywords:** Lung cancer, Proliferation, Migration, Invasion, Reactive oxygen species

## Abstract

**Background:**

Docosahexaenoic acid(DHA) inhibits tumor growth and progression in various cancers, including lung cancer. However, the mechanisms involved remain unclear. The aim of this study was to identify the mechanism of DHA in inhibiting progression of non-small cell lung cancer (NSCLC) in vitro.

**Methods:**

The proliferation of A549 was tested by MTT, and cell apoptosis was analysed using flow cytometer. The migration and invasion were examined respectively by wound healing assay and Transwell invasion assay. The level of ROS (reactive oxygen species, ROS) was checked by DCF (dichlorodihydrofluorescein, DCF) production in cells. The apoptosis associated protein (caspase-3, PARP,Bax,Bcl-2 and survivin) and metastases associated proteins including HEF1, MMP9 and VEGF were detected by Western blot, and the same method was used in the expression of PI3K and Akt.

**Results:**

DHA inhibited proliferation and induced apoptosis of A549 cells. Moreover, it suppressed the invasion and metastasis of A549 cells, while downregulating the levels of metastasis-associated proteins, including HEF1, matrix metallopeptidase (MMP9), and vascular endothelial growth factor (VEGF), in a dose -dependent manner. In addition, DHA inactivated Akt phosphorylation. All of these responses were associated with the accumulation of intracellular ROS. DHA downregulated the level of antioxidant enzymes such as catalase, while the antioxidant N-acetyl-cysteine (NAC) reversed the effect of DHA, which further validated our findings.

**Conclusions:**

The present study demonstrates that DHA inhibits the development of non-small lung tumors through an ROS-mediated inactivation of the PI3K/Akt signaling pathway.

## Background

Despite the extensive improvements in biotechnology, lung cancer treatment has not significantly improved. For decades, lung cancer has remained the most common cancer and the main cause of cancer-related deaths, in China and the US [1, 2].

Approximately 80% of lung cancer cases involve non-small cell lung cancer.

(NSCLC) [3]. The survival of NSCLC patients after therapy remains relatively low due to cancer metastasis. Furthermore, metastasis of NSCLC at the time of diagnosis has increased from 30%–40% [4, 5] to 47.3% [6] in the past decade. Moreover, these cancers recur in approximately 45% of NSCLC patients after complete resection, and is mainly caused by metastasis (73.4%) [7]. Therefore, the prevention and treatment of metastatic lung cancer are of great importance. Therapeutic regimens for NSCLC with metastasis is mainly dependent on the mode of metastasis [6]. Although strategies for chemotherapy, radiotherapy, and targeted therapy of patients have improved in recent years, these could not be extensively applied due to side effect and drug resistance. Several natural products and non-toxic agents that regulate the pathogenesis of cancer have been studied. Dietary fish oil (FO) has been demonstrated to have beneficial effects on various types of cancer [8–10]. The main components of FO that affect cancer cells are docosahexaenoic acid (DHA) and eicosapentaenoic acid (EPA), which have been demonstrated to improve tumor toxicity, reduce off-target toxicity, protect off-target tissues [8], and have no adverse effects [11]. In particular, DHA decreases proliferation and induces apoptosis in various cancer cells, including NSCLC [12–16].

However, whether DHA inhibits the metastasis and invasion of NSCLC cells remain unclear. Although DHA inhibits cancer cell migration and invasion in prostate cancer [17], breast cancer [18, 19], colorectal cancer [20], renal cancer [21], its underlying molecular mechanism remains elusive. Moreover, the mechanism by which DHA inhibits tumor cell growth apparently differs among tumors. Some research studies have demonstrated that DHA induces cancer cell apoptosis via reactive oxygen species (ROS) production in prostate cancer [22], and colon cancer [23, 24]. However, a few studies have also suggested that DHA inhibits cancer cell growth by inducing oxidative stress in liver cancer [25]. Further research is thus required to elucidate the effect of DHA on specific cancer cells.

The aim of the present study was to investigate the effect of DHA on the cell growth, migration, and invasion of the NSCLC cell line A549, while also exploring DHA’ s possible mode of action.

## Materials and methods

### Chemicals and reagents

DHA, BSA, MTT and Annexin V/PI were purchased from Sigma-Aldrich (St. Louis, MO, USA). RPMI-1640 and Eagle’s minimal essential medium (EMEM), fetal bovine serum (FBS), penicillin, streptomycin, L-glutamine, carboxy-H2DCFDA and oxidized carboxy-DCFDA were from Invitrogen (Carlsbad, CA, USA). The antibodies against VEGF, MMP2, MMP9, SOD1, and β-actin were obtained from Santa Cruz (Santa Cruz, CA, USA). The antibodies against HEF1, Akt, p-Akt, caspase-3, and cleaved PARP1 were purchased from Cell Signaling Technology (Danvers, MA, USA). The antibody against SOD2 was purchased from Millipore (Billerica, MA, USA). The antibody against catalase was obtained from Novus Biologicals (Littleton, CO, USA).

### Cell culture

The NSCLC cell line A459 was obtained from the American Type Culture Collection (Rockville, MD, USA) and cultured in RPMI-1640 or DMEM supplemented with 10% heat-inactivated FBS, 100 units/mL penicillin, 100 μg/mL streptomycin, and 1% L-glutamine. The cells were maintained at 37 °C in a humidified 5% CO_2_ atmosphere incubator and sub-cultured twice a week. In MTT, A549 cells were treated with DHA at concentrations of 6.25, 12.5, 25, 50, 75, 100 μM for 24 h; in other experiments, A549 cells were treated with DHA at concentrations of 25, 50, 75 μM for 24 h. DHA was first dissolved in ethanol to 50 mM, and then diluted using culture medium. Cells were treated with the same volume of ethanol were used as the solvent control.

### Cell viability assay

The cytotoxic activity of DHA was measured based on cell viability as determined by the 3-(4,5-dimethylthiazol-2-yl)-2,5-diphenyl tetrazolium bromide (MTT) assay. The logarithmically growing A549 cells were plated onto a flat-bottom 96-well microplate at a density of 1 × 10^4^ and cultured for 24 h, and then treated with DHA. Absorbance was measured at 570 nm using a multi-well plate spectrophotometer. The results were calculated as the percentage of absorbance with respect to that of the control. All experiments were performed in triplicate.

### Colony formation assay

The A549 cells were seeded in triplicate (500 cells per 60-mm culture dish) with DHA or without DHA and incubated at 37 °C for 2 weeks to form clones. Then, the cells were washed with PBS, fixed with 4% paraformaldehyde for 10 min, stained with 1% crystal violet for 30 min and observed under a light microscope. The number of colonies was counted, and cell survival was expressed as the fold change in the number of cells compared to that in the control cells.

### Apoptosis analysis

Apoptosis in A549 cells was measured using an Annexin V-fluorescein isothiocyanate (FITC)/propidium iodide (PI) double staining kit according to the manufacturer’s protocol. Briefly, A549 cells were grown in 10-cm dishes with RPMI 1640 medium. Upon reaching 70%–80% confluency and following serum starvation overnight, the cells were treated with either DHA or PBS. After 24 h, the cells were washed with PBS, digested with 0.25% trypsin/EDTA, and washed with PBS. The cell suspension was then centrifuged, and then the cells were re-suspended in 500 μl 1 × binding buffer containing 5 μL of Annexin V-FITC and 10 μL of PI. After incubation in the dark at room temperature for 10 min, the cells were measured by flow cytometry.

### Wound healing migration assay

To investigate cell migration, the A549 cells were allowed to grow to 70%–80% confluency in 6-well plates. To inactivate cell proliferation, the cells were starved overnight with serum free medium. The cells were then scraped with a 200 μL pipette tip and washed twice with PBS, and treated with RPMI 1640 media containing DHA at the indicated concentrations. The cells were incubated at 37 °C for 24 h and imaged. Cell migration was then quantified by manual counting the number of cells within the scraped region relative to that of the control.

### Boyden chamber invasion assay

Boyden chambers consisting of a 24-well cell culture insert membrane filter (8-μm pore size) (Cell Biolabs, Inc., CA, USA) was used to determine cell invasion. Briefly, the top surface of the chamber was coated with 100 μL of Matrigel® (100 μg/mL). Then the upper chambers were seeded with 1 × 10^5^ cells/well in 100 μL of RPMI 1640 (serum free), and the bottom chambers were filled with 500 μL of RPMI 1640 supplemented with 10% FBS as attractant. Both media contained the indicated concentrations of DHA. After 24 h, the gel and cells within the upper compartment of the insert were gently removed using a cotton swab. The cells spreading on the bottom side were fixed with cold 4% paraformaldehyde and stained with crystal violate. The number of cells in randomly selected regions of the chamber was counted under a microscope.

### Measurement of intracellular ROS

To determine the level of intracellular ROS generation, carboxy-H2DCFDA (Invitrogen) and oxidized carboxy-DCFDA (Invitrogen) were used. Cells were seeded in a 24-well plate and allowed to attach overnight. Then, the cells were treated with DHA for 24 h. Control cells were treated with ethanol (0.1% in medium). Each treatment was performed in triplicate.

The cells were washed with PBS twice and incubated with 10 μM of H2DCFDA and DCFDA separately in the serum-free medium for 30 min at 37 °C. Then, the cells were washed with PBS twice and analyzed by using a SPECTRA max Gemini XPS plate reader. The fluorescence intensity of DCF was measured at a wavelength of 492 nm (excitation) and wavelength of 517 nm (emission). The results were normalized to that of H2DCFDA and DCFDA (the ratio of H2DCFDA/DCFDA), and presented relative to that of the control.

### Western blot analysis

Whole cell extracts of the A549 cells were lysed in RIPA buffer containing a protease inhibitor cocktail. The protein concentration of all samples were measured by using the Bradford protein assay. Proteins were separated by SDS-PAGE and transferred to nitrocellulose membranes. The membranes were incubated with primary antibodies (dilution of each antibody was provided by the manufacturer) followed by incubation with secondary antibodies (dilution is 1:10,000) conjugated to horseradish peroxidase (HRP). Immuno-reactive proteins were detected by using the enhanced chemiluminescence reagent (Amersham). Then, 10 mM NAC [26] was added or not added prior to DHA treatment.

### Statistical analysis

All data were expressed as the mean ± standard deviation (SD). Statistical analysis was conducted using the student’s *t*-test by SPSS 13.0. A *p* value ≤0.05 was considered statistically significant.

## Results

### Effect of DHA on A549 cell viability

**Fig. 1:**
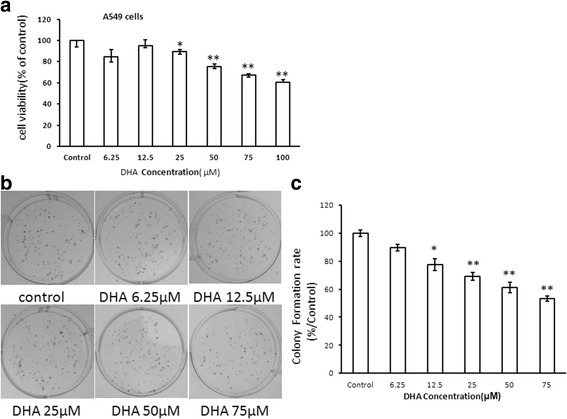
DHA plays a crucial role in suppressing the proliferation of A549 cells. MTT assay (**a**) and colony formation assay (**b**, **c**) show a decrease in growth rate in DHA-treated cells compared to that in the control. The absorbance was normalized to that of the control (100%). The number of colonies was quantified in the colony formation assay. Each bar represents the mean ± SD of three independent experiments. *P < 0.05, **P < 0.01

To investigate the effect of DHA on the proliferation of NSCLC cells, the MTT cell viability assay was performed using the A549 cells, and the colony formation assay was conducted on the A549 cells. Results showed that DHA reduced cell proliferation (Fig. 1a) at the concentration of 25 μM, and decreased cell growth from 50 μM dramatically. The colony formation assay displayed a two-fold decrease in the colony number of A549 cells after treatment with 75 μM DHA relative to that in the control (Fig. 1b and c).

### DHA induces apoptosis in A549 cells

**Fig. 2:**
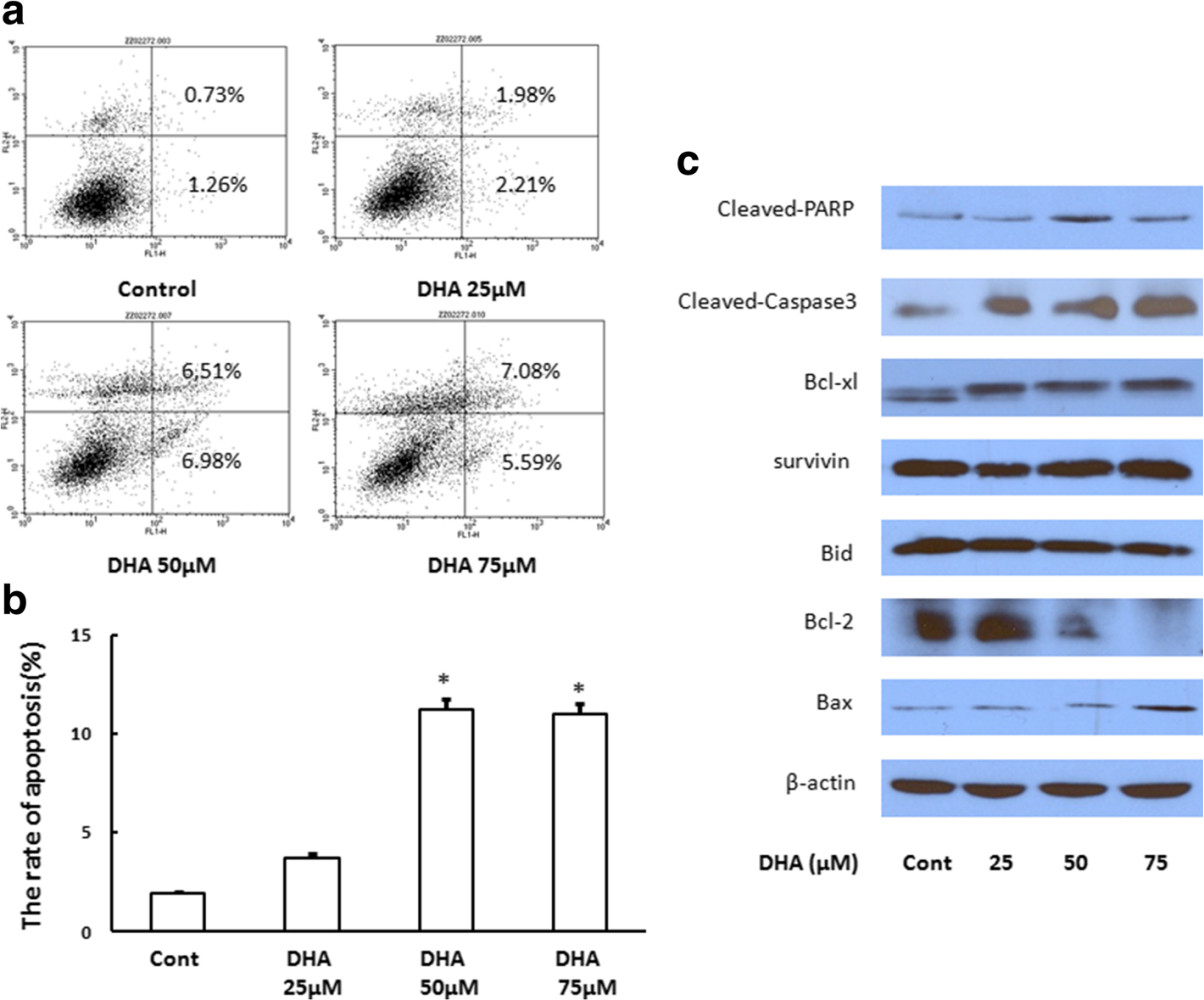
DHA induces the apoptosis in A549 cells. The rate of apoptotic cell death increased in the presence of 50 μM and 75 μM DHA (Fig. 2**a** and **b**). The level of the cleaved fragment of PARP slightly increased, whereas that of caspase3 was significantly elevated. The level of Bcl-2 decreased dramatically and that of Bax increased slightly (Fig. 2**c**)

No difference in apoptotic rates were observed between cells exposed to 25 μM DHA and the control. However, the application of 50 μM DHA resulted in an increase in the apoptotic rate of A549 cells. The early apoptotic rate reached 6.98% and late apoptotic rate was 6.51% in 50 μM DHA-treated cells, whereas there was no difference between cells treated with 50 μM DHA and 75 μM DHA (Fig. 2a and b). These two groups were evidently different from the control. Western blot analysis showed that the level of the cleaved poly-ADP-ribose polymerase (PARP) protein slightly increased, whereas that of caspase 3 significantly increased following DHA treatments. No changes in the expression of Bcl-xl, survivin, and Bid were observed, whereas that of Bcl-2 markedly decreased with 50 μM and 75 μM DHA in a dose-dependent manner. However, the expression of Bax increased slightly in 75 μM DHA group (Fig. 2c).

### DHA decreases the migration and invasion of A549 cells

**Fig. 3:**
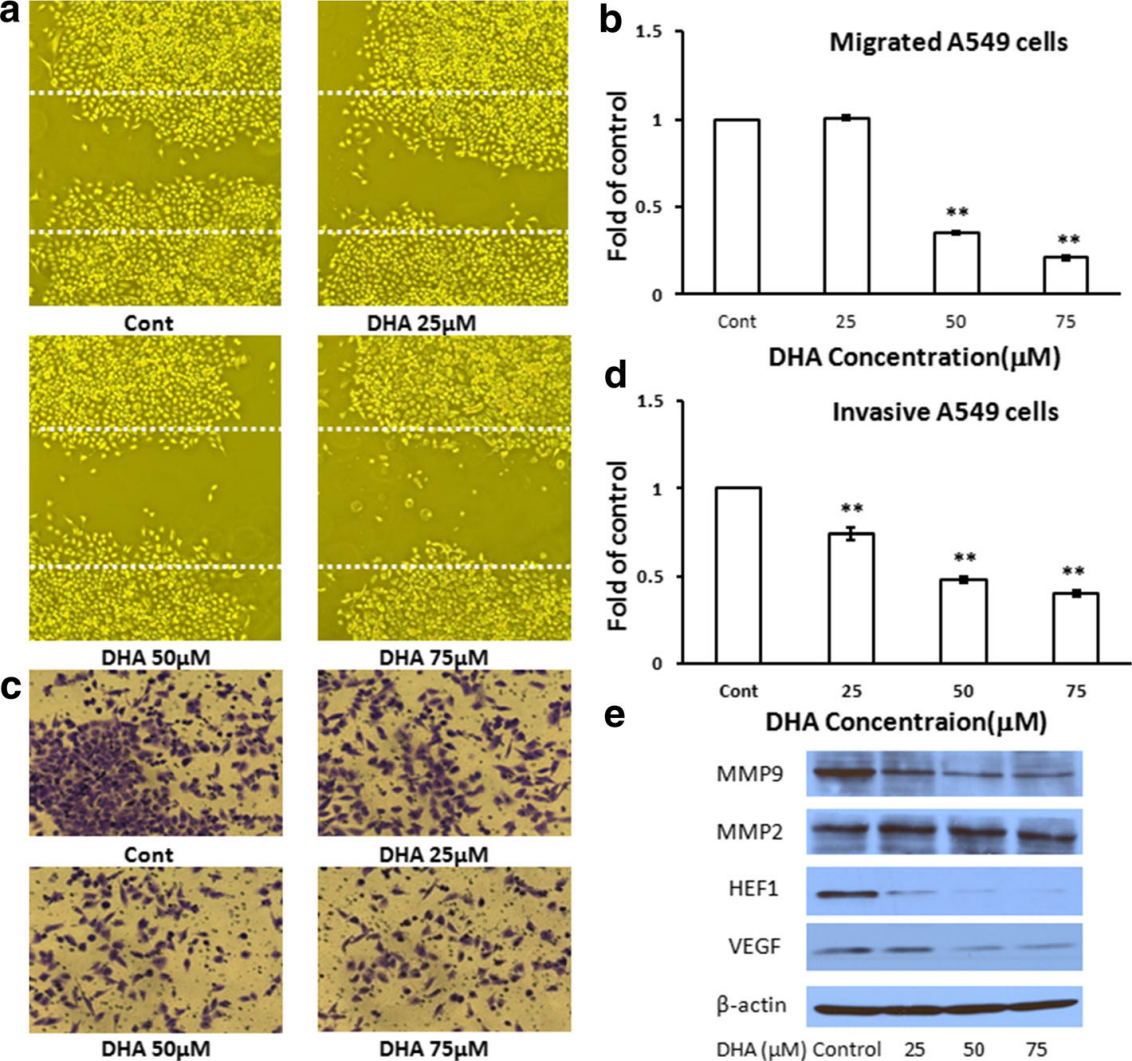
DHA decreased the migration and invasion capacity of A549 cells. The application of DHA induced a significant reduction in the migration (Fig. 3 **a** and **b**) and invasion (Fig. 3 **c** and **d**) of A549 cells relative to that in the control. *p < 0.05, **p < 0.01 vs. vehicle control. The levels of MMP9, HEF1 and VEGF were reduced significantly by DHA (Fig. 3 **e**)

The effect of DHA on A549 cell migration was tested by using the wound healing migration assay. After treatment with DHA at the indicated concentrations for 24 h, images of the migratory cells were captured and used in cell counting. DHA treatment of A549 cells resulted in a significant inhibition of cell migration from the concentration of 50 μM to 75 μM (Fig. 3a and b). The effect of DHA on cell invasion was also assessed by using a modified Boyden chamber that was coated with Matrigel®. The results showed that DHA treatment suppressed the invasion of A549 cells from 25 μM to 75 μM (Fig. 3c and d). The expression of invasion and migration- associated proteins such as MMP9, HEF1, and VEGF were suppressed by DHA. However, there was no change in the expression of MMP2 (Fig. 3e). These findings indicate that DHA effectively inhibits NSCLC progression.

### DHA triggers the generation of ROS and inhibits Akt phosphorylation

**Fig. 4:**
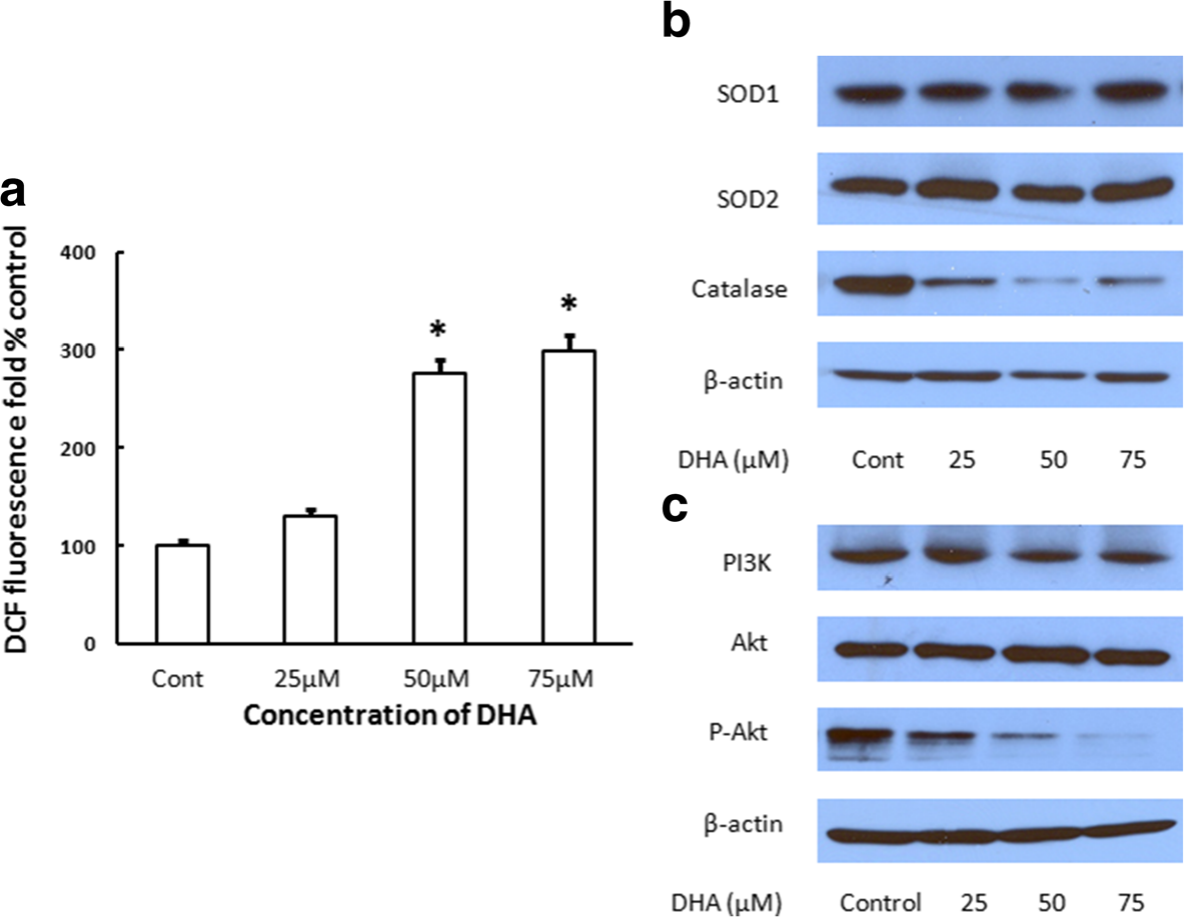
DHA increased the generation of ROS and inhibited the phosphorylation of Akt. The generation of ROS decreased with 50 μM and 75 μM, following a dose- dependent manner (Fig. 4 **a**). In terms of antioxidant enzymes, the expression of catalase significantly decreased (Fig. 4 **b**). DHA decreased the expression of PI3K slightly and markedly reduced the level of Akt phosphorylation (Fig. 4 **c**)

To determine the role of ROS in DHA-induced apoptosis and NSCLC progression. ROS generation was examined based on the analysis of DCF signals. Fluorescence intensity produced by DCFDA was enhanced in a dose-dependent manner and was significantly higher than that of the control group (Fig. 4a). To further investigate the mechanism underlying the increase in the rate of ROS generation, antioxidant enzymes and the Akt signaling pathway were evaluated. No changes in the SOD1 and SOD2 levels were observed after the cells were exposed to DHA, whereas that of catalase expression was downregulated by DHA, particularly, at the concentration of 75 μM (Fig. 4b). DHA exposure resulted in a slight decrease in the expression of PI3K and dramatic inhibition of the phosphorylated Akt (Fig. 4c).

### NAC reversed the effect of DHA on A549 cells

**Fig. 5:**
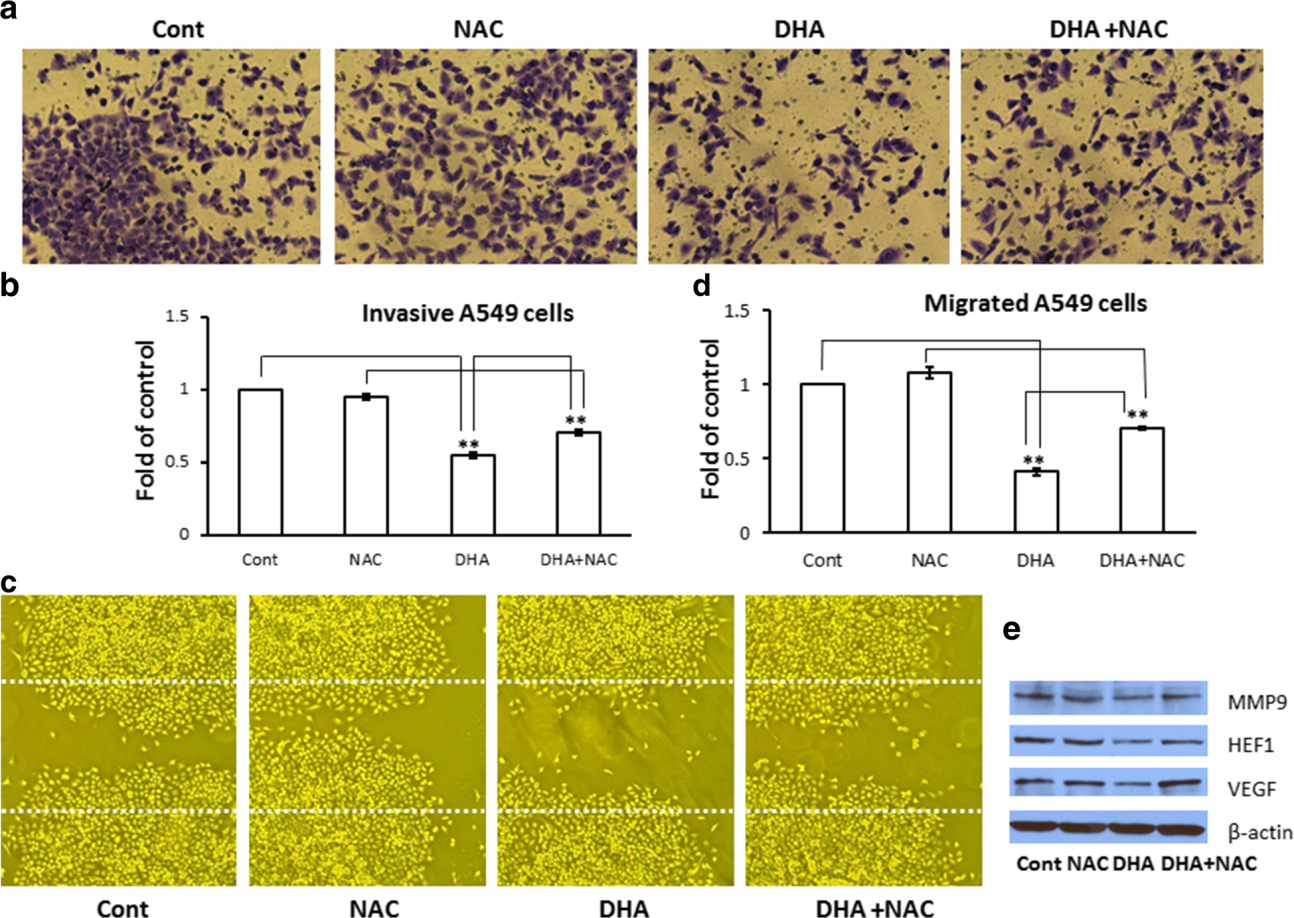
The effect of DHA on A549 cells was reversed by NAC. Invasion and migration of A549 cells with or without 10 mM NAC (Fig. 5 **a** and **c**). NAC significantly reversed the effect of DHA (Fig. 5 **b** and **d**), *p* < 0.05. The expression of MMP9, HEF1, and VEGF was upregulated by NAC (Fig. 5 **e**). DHA-induced apoptosis of A549 was slightly reversed by NAC

To further verify the role of ROS in the mechanism of action of DHA, A549 cells were exposed to the ROS scavenger, NAC, alone or in combination with DHA for 24 h. Results showed that NAC reversed partially the DHA-induced reduction in the invasion and migration of A549 cells (Fig. 5a-d). The expression of MMP9, HEF1, and VEGF was also reverted by NAC (Fig. 5e).

## Discussion

The anticancer properties of numerous natural products including dietary fish oil have been investigated in prostate cancer [22, 27–29], breast cancer [18, 29], skin cancer [28], and lung cancer [10, 30, 31]. In addition, fish oil supplementation enhances the effects of chemotherapy in colorectal cancer [32, 33]. The main component of fish oil consists of ω-3 polyunsaturated fatty acids (PUFAs) such as DHA and EPA. The beneficial effect of fish oil is apparently due to specific concentration of DHA, which inhibits the growth of cancer cells. The present study showed that DHA inhibited the proliferation of A549 cells starting with a concentration of 25 μM, and the extent of inhibition increased in a dose-dependent manner. All these results demonstrated the therapeutic role of DHA.

Previous studies have investigated the effect of DHA on the induction of apoptosis [31, 34], invasion and metastasis [17, 18, 35] of cancer cells. However, the exact molecular mechanism underlying these properties of DHA on cancer cells remains unclear. Studies on the mechanism of DHA in lung cancer are limited. The present study thus investigated the effect of DHA on A549 cell apoptosis, and cancer progression, including their underlying mechanisms.

Caspase-3 is an apoptosis effector enzyme that is triggered by intrinsic (mitochondria) and extrinsic pathways. Our study showed that DHA activates Caspase-3 and degrades another apoptosis marker, PARP. In the intrinsic apoptosis pathway, Bax transfers to the cell membrane, and binds to Bcl-2, thereby protecting the mitochondrial membrane, which in turn leads to the release of cytochrome c, and ultimately resulting in apoptosis. Our results showed that DHA downregulates Bcl-2 and upregulates Bax, whereas that of Bcl-xl and Bid did not change. These results suggest that DHA initiates the apoptosis cascade by regulating Bax and Bcl-2 in A549 cells which coincides with the results of other studies [36, 37]. In contrast to the findings of Sam et al. [38], the level of survivin did not change, which may be attributable to the fact that the effect of DHA varies among different types of cancer.

Previous studies have shown that DHA inhibits tumor growth and induces apoptosis through oxidative stress [22–24, 39], and SOD1 plays an important role in determining the cytotoxic effects of DHA on different tumor cells [40]. However only a few studies have shown that DHA suppresses cancer growth by inducing antioxidative effects [25, 41]. In addition, some studies have indicated that DHA inhibits tumor growth, metastasis, and angiogenesis through the production of DHA metabolites [42].

The findings of the present study demonstrate that DHA not only induces apoptosis but also inhibits tumor cell invasion and metastasis via ROS production. Although no changes in the level of SOD1 and SOD2 were observed, catalase expression decreased after DHA treatment. A previous study has suggested that DHA increases mitochondrial phospholipid unsaturation to upregulate ROS [23]. The present study demonstrated that DHA increases ROS production by downregulating catalase. In line with these results, antioxidant NAC reversed the effects of DHA on A549 cells, which further supports our findings.

Several studies have shown that DHA can induce apoptosis in cancer cells by activating the MAPK signaling pathway, and suppressing Akt phosphorylation [43–47]. Our results showed that DHA induces apoptosis by inhibiting the phosphorylation of Akt at serine 473.

Several factors are involved in NSCLC progression, including MMP9 [48–50], HEF1 [51] and VEGF [52, 53]. The present study utilized MMP-9, HEF1, and VEGF as indicators of the therapeutic effect of DHA on A549 cells. Our results showed that the application of DHA resulted in significant reduction in the expression of MMP-9, HEF1 and VEGF, thereby suggesting that DHA suppresses metastasis and invasiveness of A549 cells by inhibiting the expression of MMP-9, HEF1 and VEGF.

Taken together, the findings of present study indicate that DHA inhibits A549 cell growth, migration and invasion via the accumulation of intracellular ROS. ROS inactivates the PI3K/Akt pathway, which in turn inhibits the growth and development of cancer. These results demonstrate that DHA should be considered as a potential preventive agent for the recurrence and metastasis of postoperative NSCLC. Nevertheless, the mechanism of DHA in different cancers requires further investigation.

## Conclusions

In conclusion, DHA not only induces the apoptosis of NSCLC cells in vitro, but also suppresses the migration and invasion of this cells. Meanwhile, DHA undergoing anticancer effect on non-small lung tumors through an ROS-mediated inactivation of the PI3K/Akt signaling pathway.

## Abbreviations

DCF: Dichlorodihydrofluorescein
DHA: Docosahexaenoic acid
EMEM: Eagle’s minimal essential medium
EPA: Eicosapentaenoic acid
FBS: Fetal bovine serum
FITC: Fluorescein isothiocyanate
FO: Dietary fish oil
HRP: Horseradish peroxidase
MMP9: Matrix metallopeptidase
MTT: 3-(4,5-dimethylthiazol-2-yl)-2,5-diphenyl tetrazolium bromide
NAC: N-acetyl-cysteine
NSCLC: Non-small cell lung cancer
PI: Propidium iodide
PUFA: Polyunsaturated fatty acids
ROS: Reactive oxygen species
SD: Standard deviation
VEGF: Vascular endothelial growth factor
